# Risk management in a blood center: A step towards risk reduction

**DOI:** 10.6026/973206300221263

**Published:** 2026-02-28

**Authors:** Sanjay Kumar Thakur, Anil Kumar Sinha, Santosh Kumar Sharma, Arun Kumar, Lokesh Kumar, Aarzoo Jahan, Ruchika Gupta, Sompal Singh

**Affiliations:** 1 Department of Blood Bank, Regional Blood Transfusion Centre, Hindu Rao Hospital and NDMC Medical College, North Delhi 110007, Delhi, India; 2 Department of Zoology, VeerKunwar Singh University Ara, Bihar, India; 3 Department of Pathology, Hindu Rao Hospital and NDMC Medical College, North Delhi 110007, Delhi, India; 4 Department of Cytopathology, ICMR-National Institute of Cancer Prevention and Research, Noida 201301, Uttar Pradesh, India

**Keywords:** Risk management, risk priority number (RPN), blood bank, hem vigilance systems, likert scale, catastrophic

## Abstract

Given the central role of blood bank in patient care, any risks within involving blood bank can have serious implications, potentially affecting patient outcomes. A systematic approach is presently lacking in risk management of blood centers. Therefore, it is of interest to apply techniques to implement effective risk management strategies in a blood bank, with the goal of minimizing its occurrence and enhancing overall safe transfusion. The probability of each risk event was rated on a five-point Likert scale: Very Low (1), Low (2), Moderate (3), High (4) and Very High (5). The severity of potential outcomes was similarly rated: Negligible (1), Minor (2), Moderate (3), Severe (4) and Catastrophic (5). These scores were used to calculate the unweighted Risk Priority Number (RPN) for each risk and risks with higher RPNs were prioritized for mitigation efforts. The top 10 risk with highest RPN seen in our study were: donor send without proper counselling, blood request form not filled properly, misbehaviour by attendants, wrong blood group on blood bag, wrong labelling of blood bag/sample, request from professional donor, blood bag labelling mistake, donor standing quickly after blood donation, RBC contamination in platelets preparation, sample insufficient for analysis.

## Background:

Risk management is a systematic approach for identifying, assessing and prioritizing risks to minimize their impact, particularly in critical environments like blood centres, where errors can severely affect patient safety [1]. Blood centers are vital for providing blood products for emergencies and routine treatments, yet they face numerous risks at every stage of the blood supply chain, including collection, processing, storage, distribution and transfusion [2, 3]. Effective risk management in these settings is essential to protect both blood donors and recipients from adverse outcomes. The blood supply chain involves a series of interconnected processes that are highly susceptible to errors, especially considering factors like blood type compatibility [4, 5] and the short shelf life of blood products [6]. A single mistake in any stage, such as incorrect identification or compatibility errors, can result in life-threatening consequences [1, 2]. Despite improvements in specific stages of the process, such as hemovigilance systems and traceability technologies, logistical risks during blood distribution and handling remain underexplored [7]. Several factors, including environmental conditions, staff training and human error, contribute to the risks within the blood supply chain [8]. Most studies on blood centers focus on specific stages, such as blood collection or storage, with less emphasis on the overall risk management across the entire supply chain. Research has shown that errors in blood ordering, labelling and handling contribute significantly to transfusion-related risks [2, 9]. However, upstream processes and logistics, often overlooked, can also lead to substantial safety issues in the transfusion process [10].

Currently, there are several guidelines available to assist laboratory managers in the implementation of risk management practices, including "CLSI EP18-A2-Risk Management Techniques to Identify and Control Error Sources," "CLSI EP23-A-Laboratory Quality Control Based on Risk Management" and "ISO/TS 22367:2020-Medical Laboratories-Reduction of Error Through Risk Management and Continual Improvement" [11]. Additionally, Standards such as the American Association of Blood Banks (AABB) Technical Manual emphasize the necessity of implementing quality management systems that encompass risk management strategies across all operational aspects of blood banking [12]. Similarly, ISO 31000:2009 defines risk as the "effect of uncertainty on the achievement of objectives," underscoring the importance of identifying and addressing risks proactively to ensure operational success [13]. ISO 9001:2015 further stresses the importance of planning actions to manage risks and opportunities, ensuring continuous improvement in quality management. However, these guidelines are not mandated by accreditation bodies, except in relation to quality control procedures. Consequently, risk management is often not implemented as a systematic or comprehensive approach in clinical laboratories [11]. In clinical histocompatibility testing, risk assessment and mitigation are now critical components of a laboratory's QMS [14]. Therefore, it is of interest to systematically identify risks at each stage of the blood supply chain, enhancing transfusion safety and improving patient outcomes.

## Materials and Methods:

## Ethical statement:

The present study was conducted after approval from the Institutional Review Board (IRB) of North DMC Medical College and Hindu Rao Hospital, Delhi-7, by approval No: IEC/NDMC/2021/69. As no additional blood samples of donors or patient was used for this study, hence separate informed consent was not required.

## Study design:

This study used a cross-sectional observational design to identify and assess risks in blood banking operations. The study was carried out at the Regional Blood Transfusion Centre situated inNorth DMC Medical College and Hindu Rao Hospital, Delhi-7.

## Inclusion and exclusion criteria:

Not applicable.

The study's methodology was carried out in three stages: 1. Risk Identification, 2. Risk Assessment and 3. Risk Reduction.

## Risk identification:

Risk identification was carried out through three structured brainstorming sessions with a multidisciplinary team, including blood bank staff, doctors and management personnel. Risks were categorized and identified across various sections of blood bank including: Donor screening and recruitment, Blood collection and processing, Blood storage and transportation, Blood transfusion and component preparation and tabulated.

## Risk assessment:

The identified risks were evaluated using the Likert scale for both probability (p) and impact (i), as outlined below:

## Probability of occurrence:

Based on observations made by blood bank employees over a ten-year period, the likelihood occurrence of each risk was estimated. The number of events during a ten-year period divided by 10x365 days was used to compute the daily probability.

## Impact score:

By using employee brainstorming, the possible impact of the risk was categorized semi-quantitatively based on the likelihood that it would interfere with the service of blood bank. Their severity impact scores are divided into the following categories: Negligible (1), Minor (2), Moderate (3), Severe (4) and Catastrophic (5).

## The risk priority number (RPN):

RPN was calculated by multiplying the probability and impact for each identified risk as follows: Risk Priority Number (RPN) = Probability of occurrence x Impact score.

## Risk reduction:

Based on the RPN, the risks were prioritized. The Top Ten Risk with high risk Scores (RPN) were selected and categorized as those with highest priority and others were categorized into moderate and low risk priority. The mitigation strategies were developed accordingly, focusing on the most critical risks with the highest RPNs.

## Results and Discussion:

A comprehensive risk analysis was conducted across various functional sections of the blood bank to identify, quantify and prioritize risks associated with blood bank operations, by using a structured approach. A total of 55 risks (Table 1) were identified during the brainstorming sessions, with the likelihood and impact of each risk assessed using a five-point Likert scale. The identified risks were categorized into several key sections: Donor Screening and Blood Collection, Blood Component Preparation Lab, TTI Lab, Main Lab, Reception Counter, Natural Disaster, Environmental Risks, Manpower and Laboratory Hazards. The Donor Room exhibited the highest number of identified risks (17 in total), with "Donor sent to blood without proper counseling" presenting the highest RPN (2.959), indicating a high probability (0.986) and moderate impact (3). Other significant risks included "Blood request form not filled properly" (RPN: 2.137) and "Misbehavior by patients attendant" (RPN: 1.140). Although several risks such as "Blood splatter", "Incorrect venipuncture site asepsis" and "Skipped post-donation counseling" had very low RPNs (≤0.033), their cumulative presence contributes to the overall risk profile of this section. Eight risks were recorded in the Component Lab. The most prominent was "RBC contamination" (RPN: 0.570), with a moderate probability (0.285) and low impact (2). Equipment failures like "Centrifuge machine not working" and "Blood bag leakage" both had equal RPNs (0.164), suggesting moderate impact but low occurrence. Most other equipment-related risks such as freezer temperature anomalies and agitator malfunction had minimal RPNs (0.014), reflecting their infrequent occurrence. Three risks were identified in the TTI Lab, with uniformly low RPN values (≤0.014). Notably, "Skip isolation of TTI reactive blood from quarantine" and "Human error during result interpretation" was both calculated at RPN 0.014, suggesting low probability but high potential impact. The Main Lab reported six risks. Among these, "Wrong blood group label on blood bag" held the highest RPN (1.140), denoting moderate probability (0.285) and high impact (4). Labeling errors (RPN: 0.712) and blood spills (RPN: 0.427) followed, while rare events like "Sample lost or not found" had a minimal RPN (0.022).This area had six identified risks. "Sample insufficient for analysis" and "Improperly labeled sample" presented the highest RPNs (0.570 and 0.493, respectively). Other issues like "Hemolysed samples" and equipment failures (e.g., "Hb cuvette machine not working") were lower in RPN but still relevant. Environmental factors such as "No electricity supply" and "A.C. not working" each showed RPNs of 0.099, reflecting low to moderate impact with low probability. Civil issues and lack of water supply had minimal scores (0.033), suggesting a lesser operational threat.

## Natural disasters:

**Figure 1:**
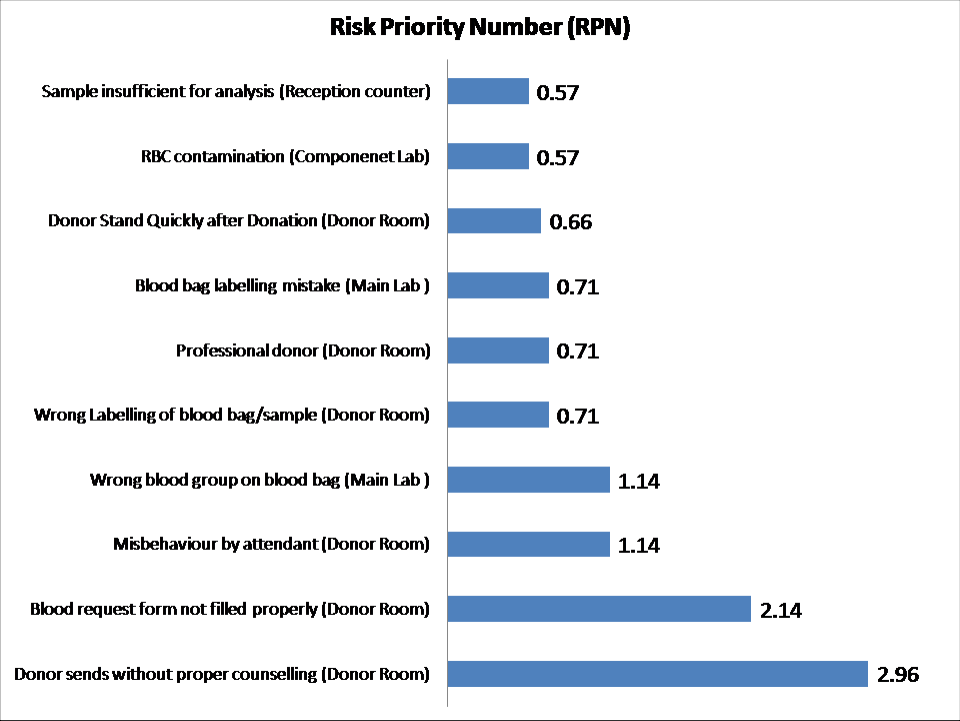
The top ten identified risks in the blood bank, based on their risk priority number (RPN).

Only "Earthquake" was identified as a natural disaster risk, with a low probability (0.005) but high impact (5), resulting in an RPN of 0.027.Personnel-related risks included "Ill health of blood bank staff" (RPN: 0.427) and "Manpower competency issue" (RPN: 0.132). Risks related to unavailability and administrative issues, such as "Strike in hospital" and "Staff transfer", had negligible RPNs (≤0.027), indicating minimal influence under normal conditions. Among laboratory hazards, "Biological hazard" had the highest RPN (0.164), followed by "Fire safety" (0.014). Mechanical and chemical hazards were reported with lower RPNs (0.011), suggesting they are rare but should still be monitored for safe laboratory operations. The distribution of RPN values reveals that the blood bank's Donor Screening and Blood Collection and Reception Counter sections carry the highest overall risks, primarily due to administrative and procedural errors (e.g., incomplete forms, improper labelling). Equipment malfunction risks in the Blood Component Preparation Lab and Main Lab, as well as TTI Lab challenges were also notable but scored lower in comparison. Environmental and manpower risks, while present, were ranked lower in severity. The top ten identified risks in the blood bank, based on their Risk Priority Number (RPN) were presented in Figure 1 and their mitigation strategy was presented in Table 2. The top 10 highest unweighted risk score in our study were: donor send without proper counselling, blood request form not filled properly, misbehaviour by attendants, wrong blood group on blood bag, wrong labelling of blood bag/sample, RBC contamination, Sample insufficient for analysis, improperly labeled sample, Blood spills and Ill health of blood bank staff. Blood bag labelling mistake, donor stands quickly after donation, RBC contamination in platelets preparation, sample insufficient for analysis. Despite significant attention to blood transfusion risks, there remains a lack of comprehensive understanding of how these risks originate and spread throughout the blood supply chain (BSC) [7]. This issue is critical because many adverse events affecting patient safety stem not from clinical activities but from the mismanagement of blood products within the supply chain [15]. On the contrary, current research primarily focuses on isolated adverse events, assessing their effects at blood use points, typically after errors occur [8, 16]. Prior research indicates that critical incidents often arise from factors like electrical errors, machine malfunctions and improper system behaviour [17]. Research has traditionally focused on equipment malfunctions as the main source of errors in blood banking, but most risks occur during extra-analytical phases, such as environmental factors, manpower-related issues and pre-analytical activities [18, 19]. This highlights the need for a broader risk management approach that accounts for all stages of blood handling and transfusion. The findings of this risk assessment highlight several critical areas within the blood bank's operational framework that require immediate attention to enhance both safety and efficiency. By evaluating the likelihood, impact and overall Risk Priority Number (RPN) of identified risks across various sections, this study provides a comprehensive understanding of the most pressing challenges and the potential consequences of failure to address these risks. The results of this assessment underscore the importance of risk management strategies to minimize adverse outcomes in blood bank operations, particularly in donor screening, blood collection, component preparation and laboratory practices.

This study systematically evaluated the operational risks within different sections of a blood bank using the Risk Priority Number (RPN) method, integrating both the likelihood and potential impact of each event. The findings highlighted significant variability in risk profiles across functional areas, with the Donor Room emerging as the most critical area, contributing 5 of the top 10 risks by RPN. The highest-risk event-donor sent without proper counseling (RPN: 2.959)-reflects a gap in donor management protocols. Inadequate counseling not only jeopardizes donor safety but also increases the risk of collecting blood from unsuitable donors, potentially compromising transfusion safety. Other high-risk events such as improperly filled blood request forms, misbehavior by attendants and blood grouping errors underscore the importance of human factors, training and process standardization in ensuring safe blood bank operations. Technological and equipment-related issues like RBC contamination and labeling errors were prominent, reinforcing the need for automation, stringent SOPs and robust quality control mechanisms. Issues in the Reception such as sample insufficiency and improper labeling further highlighted vulnerabilities in pre-analytical phases, often neglected in traditional risk models. Interestingly, although environmental and natural disaster risks were identified, their RPNs were relatively low, primarily due to low occurrence probabilities. However, their potential impact remains high, indicating the necessity for disaster preparedness protocols and contingency planning. A key observation is that many high-priority risks are preventable with modest interventions-through training, procedural updates and automation. This supports the utility of proactive risk identification and prioritization in improving overall blood bank safety. For optimal risk management, regular system maintenance and control techniques are essential, with robust checkpoints to prevent accidents. In our study, after assessing laboratory risks, we selected appropriate control measures and risk reduction strategies to ensure blood transfusion services within acceptable risk levels. In risk management, remediation strategies typically involve diversifying defence mechanisms, such as implementing multiple systems for non-conformity detection and reducing vulnerability through detection systems, causal relationship charts and enhanced education and training [20]. Studies suggest conducting causal analyses, like Pareto and Ishikawa diagrams, to develop improvement plans that include staff training, compliance checks, preventive maintenance and technical evaluations in procurement, with clear actions, timelines and responsible personnel for each stage. Mitigation strategies will be focused on improving training, developing clearer protocols and ensuring stricter supervision in areas where risks are prevalent. For example, implementing better donor post-donation observation protocols and ensuring thorough checks for labelling and documentation errors will be prioritized. Based on the results, several strategies can be recommended to reduce the most significant risks identified in this study.

## Staff training and awareness:

Regular training sessions for staff in both clinical and non-clinical areas of the blood bank should be conducted. This training should cover proper blood collection techniques, post-donation care, infection control and the importance of accurate documentation. Simulation exercises may also help in building staff preparedness for emergency situations.

## Process standardization and automation:

Automating certain aspects of blood bank operations, such as blood sample labelling, blood group testing and cross matching, can significantly reduce human error.

## Use of technology:

Integration of barcode systems, electronic forms and automated checks can significantly reduce the occurrence of manual errors.

## Maintenance and quality control:

Regular equipment maintenance and a robust quality control system ensure that the tools and machines used in blood collection and component preparation are functioning properly, reducing the risk of technical failure. Backup systems, such as generators and refrigerated storage, should be in place to minimize the impact of power failures.

## Improvement of donor care protocols:

Enhancing donor care during and after donation can help reduce the risk of adverse reactions. This includes ensuring proper donor counselling, monitoring post-donation and offering appropriate refreshments.

## Monitoring and auditing:

Routine monitoring, audits and feedback loops will help and identify areas for improvement, allowing for timely interventions before risks become more serious.By addressing these key areas, the blood bank can effectively mitigate the identified risks and improve the safety and efficiency of its operations. However, while most of the likely adverse events have been considered in this study, there are limitations in terms of the scope of potential events covered. A more comprehensive study could be conducted to explore a wider range of possible adverse events, providing a more thorough assessment and potentially uncovering additional risks that were not considered in this analysis.

## Conclusion:

This study identified the donor room, reception and main laboratory as areas with the highest risk scores. The data shows the value of structured risk management in blood banking, enabling prioritization of high-impact risks and efficient allocation of resources. The results offer a practical framework for strengthening safety protocols and improving transfusion service quality. Proactive risk management is critical in blood centers to ensure donor and recipient safety. Future efforts should emphasize continuous risk monitoring, adoption of digital systems, staff engagement and periodic post-intervention risk reassessment to sustain safety improvements.

## Author contributions:

Thakur SK, Sinha AK, Sharma SK, Gupta R and Singh S designed the research. Thakur SK conducted the literature review, data collection, analysis and manuscript preparation; Thakur SK, Sharma SK, Kumar A, Kumar L, Jahan A, Gupta R and Singh S performed statistical analysis. All authors contributed equally to the final manuscript.

## Figures and Tables

**Table 1:** Probability, impact and risk score associated with blood bank operations

**Sections of blood bank**	**Risk**	**Probability (P)**	**Impact (I)**	**Risk Priority number (RPN)**
Donor Room	Donor sends without proper counseling	0.986	3	2.959
	Blood request form not filled properly	0.427	5	2.137
	misbehavior by attendant	0.285	4	1.14
	Wrong Labeling of blood bag/sample	0.142	5	0.712
	Professional donor	0.142	5	0.712
	Donor Stand Quickly after Donation	0.132	5	0.658
	Under collection	0.164	3	0.493
	double prick during venipuncture	0.285	1	0.285
	Needle prick	0.033	5	0.164
	Donor Adverse reaction	0.033	3	0.099
	Over collection	0.033	3	0.099
	Component demand not mentioned properly	0.033	3	0.099
	Blood splatter	0.033	1	0.033
	Incorrect venipuncture site asepsis	0.003	4	0.011
	Skipped blood sample for TTI tests	0.003	3	0.008
	Skipped in post donation concealing	0.003	2	0.005
	Refreshment not available/ skipped	0.003	1	0.003
Component Lab	RBC contamination	0.285	2	0.57
	Centrifuge Machine not working	0.033	5	0.164
	Blood bag leakage	0.033	5	0.164
	Blood bag tube sealer not working	0.005	3	0.016
	Deep freezer temperature	0.003	5	0.014
	Component prepared late	0.003	5	0.014
	Platelet agitator not working properly	0.003	5	0.014
	BB Refrigerator temperature	0.003	5	0.014
TTI lab	Skip isolation of TTI reactive blood from quarantine	0.003	5	0.014
	Human error during interpretation of results	0.003	5	0.014
	Maintenance of KIT temp	0.003	4	0.011
Main Lab	Wrong blood group on blood bag	0.285	4	1.14
	Blood bag labeling mistake	0.142	5	0.712
	Blood spills	0.142	3	0.427
	Patient Blood group discrepancy	0.055	4	0.219
	Patient Blood group component not available	0.066	2	0.132
	Sample lost or not found	0.005	4	0.022
Reception counter	Sample insufficient for analysis	0.142	4	0.57
	Blood sample Improperly labeled	0.099	5	0.493
	Hemolysed sample	0.033	4	0.132
	Hb cuvette Machine not working	0.033	1	0.033
	Sample collected in the wrong container	0.003	4	0.011
	Sample damaged in transport	0.003	4	0.011
Environmental risk	No electricity supply	0.033	3	0.099
	Room temperature A.C not working	0.033	3	0.099
	No water supply	0.033	1	0.033
	Civil issues (drain issue)	0.033	1	0.033
Natural disaster	Earthquake	0.005	5	0.027
Manpower	III health of BB Staff	0.142	3	0.427
	Manpower competency issue	0.033	4	0.132
	Guard unavailable in M & E	0.008	4	0.033
	MTS unavailable in M & E	0.005	5	0.027
	Strike in hospital	0.005	3	0.016
	Staff transfer	0.001	3	0.002
Laboratory hazard	Fire safety	0.003	5	0.014
	Mechanical hazards (sharp injury)-minor	0.003	4	0.011
	Chemical hazards	0.003	4	0.011
	Biological hazard	0.033	5	0.164

**Table 2:** Top ten identified risks in the blood bank, based on their Risk Priority Number (RPN) with their mitigation strategy

**Location**	**Rank**	**Risk (RPN)**	**Description**	**Mitigation Strategy**
Donor Room	1	2.959	Donor sent without proper counseling	Introduce a mandatory checklist for pre-donation counselling to ensure consistent communication of key information. Provide regular staff training and conduct periodic audits to maintain quality and compliance. Implement a digital workflow for donor registration to enhance accuracy, efficiency and data security.
Donor Room	2	2.137	Blood request form not filled properly	Implement electronic request forms with mandatory fields to ensure completeness. Train clinicians on accurate form completion. Assign dedicated staff to verify submitted information for accuracy and compliance.
Donor Room	3	1.14	Misbehavior by attendant	Provide soft-skills and behaviour training to enhance professional interactions. Enforce a clear code of conduct supported by a disciplinary policy. Establish an anonymous reporting and feedback system to encourage accountability and transparency.
Main Lab	4	1.14	Wrong blood group label on blood bag	Ensure dual verification of blood groups to prevent errors. Use barcode identification and automated grouping systems for accuracy. Conduct root cause analysis for any mismatches to identify and correct systemic issues.
Donor Room	5	0.712	wrong labelling of blood bag/sample	Implement a barcode labelling system for accurate sample identification. Enforce a label-before-draw policy to prevent mix-ups. Conduct routine audits and competency checks to ensure adherence to protocols.
Donor Room	6	0.712	Professional Donor	Thorough clinical history taking and meticulous medical examination of donors help prevent blood collection from professional or ineligible donors.
Main Lab	7	0.712	Blood bag labelling mistake other than blood group	Implement a barcode labelling system to ensure accurate sample identification. Follow a label-before-draw policy to avoid mislabelling. Conduct routine audits and competency checks to maintain procedural compliance and staff proficiency.
Donor Room	8	0.658	Donor stands quickly after donation	Train all staff to monitor donors closely post-donation to prevent sudden movements. Establish clear post-donation observation procedures, including appropriate seating and rest periods. Inform donors about the risks of standing too quickly during the counselling session.
Component Lab	9	0.57	RBC contamination	Strictly adhere to Standard Operating Procedures (SOPs) for preparation. Provide staff re-training on proper component preparation techniques. Ensure consistent adherence to the equipment maintenance schedule.
Reception	10	0.57	Sample insufficient for analysis	Implement standard volume guides for sample collection. Provide thorough phlebotomy training for staff. Conduct sample volume checks before receiving to ensure accuracy.
